# Urinary Neutrophil Gelatinase-Associated Lipocalin Can Predict the Efficacy of Volume Expansion Therapy in Patients With Hepatitis B Cirrhosis and AKI

**DOI:** 10.3389/fphar.2022.839250

**Published:** 2022-06-15

**Authors:** Zhonghui Duan, Minjie Jiang, Xiaojie Huang, Haixia Liu, Hongwei Yu, Qinghua Meng

**Affiliations:** ^1^ Department of Emergency, Beijing Youan Hospital, Capital Medical University, Beijing, China; ^2^ Department of Hepatology, Beijing Youan Hospital, Capital Medical University, Beijing, China; ^3^ Department of Infectious Disease, Beijing Youan Hospital, Capital Medical University, Beijing, China; ^4^ Department of Intensive Care Medicine, Beijing Youan Hospital, Capital Medical University, Beijing, China; ^5^ Department of Outpatient, Beijing Youan Hospital, Capital Medical University, Beijing, China

**Keywords:** cirrhosis, kidney injury, urinary biomarker, diagnostic marker, NGAL

## Abstract

**Backgrounds:** Kidney biomarkers in urine appear to be useful in differential diagnosis between acute tubular necrosis and other types of acute kidney injury (AKI) in cirrhosis. In clinical practice, prerenal azotemia (PRA) is often distinguished from other types of AKI by volume expansion therapy. The aim of the current study was to investigate the accuracy of urinary biomarkers in the differential diagnosis between PRA and other types of AKI.

**Methods:** A total of 65 patients with hepatitis B cirrhosis were prospectively included and divided into AKI and non-AKI groups. Patients with hepatitis B cirrhosis and AKI discontinue diuretics, vasodilators, and nephrotoxic drugs and give volume expansion therapy. The efficacy was judged after 48 h of treatment. Urinary biomarkers were measured at the time of diagnosis of AKI and 48 h after volume expansion therapy. Univariate and multivariate analyses were used to identify independent risk factors for nonresponse to volume expansion therapy.

**Results:** Of the 65 patients, 49 patients with newly diagnosed AKI were screened in the study, and 16 hospitalized patients with hepatitis B cirrhosis without AKI at the same period were screened as the control group. In patients with cirrhosis and AKI, 29 (59.18%) patients were in the response group and 20 (40.81%) patients were in the nonresponse group. The mortality rate in the nonresponse group was significantly higher than that in the response group (75% vs. 13.8% *p* < 0.001). After logistic regression analysis, urinary neutrophil gelatinase-associated lipocalin (NGAL) and serum creatinine (SCr) at diagnosis of AKI showed significant association with nonresponse to volume expansion therapy. The cutoff values for SCr and urinary NGAL were 128.50 µmol/L and 90.75 ng/ml, respectively. The area under the receiver operating curve (AUC) for SCr and urinary NGAL was 0.815 and 0.831.

**Conclusion:** Elevated urinary NGAL can reflect the degree of kidney injury and is an independent risk factor for nonresponse to volume expansion therapy in patients with hepatitis B cirrhosis and AKI.

## Introduction

Acute kidney injury (AKI) is common in patients with cirrhosis, occurring in 20% of hospitalized patients (Garcia-Tsao et al., 2008). Patients with cirrhosis and AKI have a poor prognosis, with the 3-month mortality after AKI ranging from 28% to 47% (Huelin et al., 2019). In some studies, patients who had cirrhosis combined with AKI had a sixfold higher in-hospital mortality rate than patients without AKI (Tariq et al., 2020). Although patients with cirrhosis and AKI have a poor overall prognosis, viable treatments do exist, but they vary depending on the cause of AKI. In cirrhosis, prerenal azotemia (PRA), ATN, and hepatorenal syndrome-AKI (HRS-AKI) are the common causes of AKI (Angeli et al., 2019). PRA is effective for volume expansion therapy, but ATN and HRS-AKI are all ineffective for the treatment (Schrier, 2010). ATN should be treated with renal replacement therapy (Krag et al., 2007; Garcia-Tsao et al., 2008). HRS-AKI may be reversed with restoration of renal perfusion, either via vasoconstrictor therapy plus intravenous albumin or liver transplant (Wong et al., 2015; Jindal et al., 2016). Although it is important to identify the different types of AKI in patients with cirrhosis, current guidelines and expert consensus have not found biomarkers with high sensitivity and specificity in identifying the cause of AKI.

Serum creatinine (SCr) of patients with cirrhosis is affected by many factors, and it is only a marker of kidney filtration, not injury. Thus, SCr may not be used to distinguish functional from structural etiologies of AKI (Verhave et al., 2005; Slocum et al., 2012). The fractional excretion of sodium (FENa) can be used to distinguish functional from structural disease in many settings of AKI. However, patients with cirrhosis often have chronic renal hypoperfusion and have an FENa <1%, even in the absence of AKI (Angeli et al., 2019). In addition, a large number of patients with cirrhosis often used diuretics, the test has been considered unhelpful in distinguishing HRS from ATN (Salerno et al., 2007). As emerging markers of kidney injury, NGAL, IL-18, kidney injury molecule-1 (KIM-1), and L-FABP are not expressed in normal kidneys and their levels increase in the urine upon kidney injury (Yang et al., 2015; Parikh, 2017). All experts agreed on the potential role of urine biomarkers in the differential diagnosis of different types of AKI in patients with cirrhosis. Currently, some published studies found that urinary NGAL and urinary IL-18 can be used as a biomarker to identify the differentiating ATN from HRS and other causes of AKI in cirrhosis (Morelli et al., 2021).

We found that most studies used urinary biomarkers to identify the differentiating ATN from HRS-AKI in cirrhosis. However, in clinical practice internists also frequently face the challenge of differentiating between PRA from ATN and HRS-AKI when assessing hospitalized patients with AKI. In clinical practice, PRA is often distinguished from other types of renal injury by volume expansion therapy, which often leads to diagnostic delays in differentiating ATN and (or) HRS-AKI from PRA. Therefore, the addition of urinary biomarkers to the clinical panel could potentially enhance clinician accuracy in distinguishing between PRA and other types of AKI (Parikh, 2017). Thus, we report the results of a prospective study performed in patients with cirrhosis and AKI to assess the biomarker with the greatest accuracy in the differential diagnosis between PRA and other types of AKI.

## Methods

### Patient Population

This is a prospective study performed in patients with hepatitis B cirrhosis admitted at Beijing Youan Hospital, Capital Medical University between September 2014 and January 2017 who had AKI either at admission or developed it during hospitalization. The diagnosis of cirrhosis was based on a combination of clinical, biochemical, ultrasonography, endoscopy, and/or histological findings.

Inclusion criteria included a known diagnosis of hepatitis B cirrhosis (see “Definitions”), age ≥18 years, and newly diagnosed AKI (see “Definitions”). Exclusion criteria included chronic kidney disease (baseline creatinine >4.0 mg/dl); acute or chronic renal replacement therapy within 2 days of enrollment; absence of baseline SCr; inability to collect a qualifying urine specimen and serum specimen within 2 days of AKI diagnosis; less than 2 days of follow-up after AKI diagnosis; and inability to comply with the clinical study protocol. Patients with hepatitis B cirrhosis who were hospitalized during the same period without AKI were selected as the control group.

**FIGURE 1:**
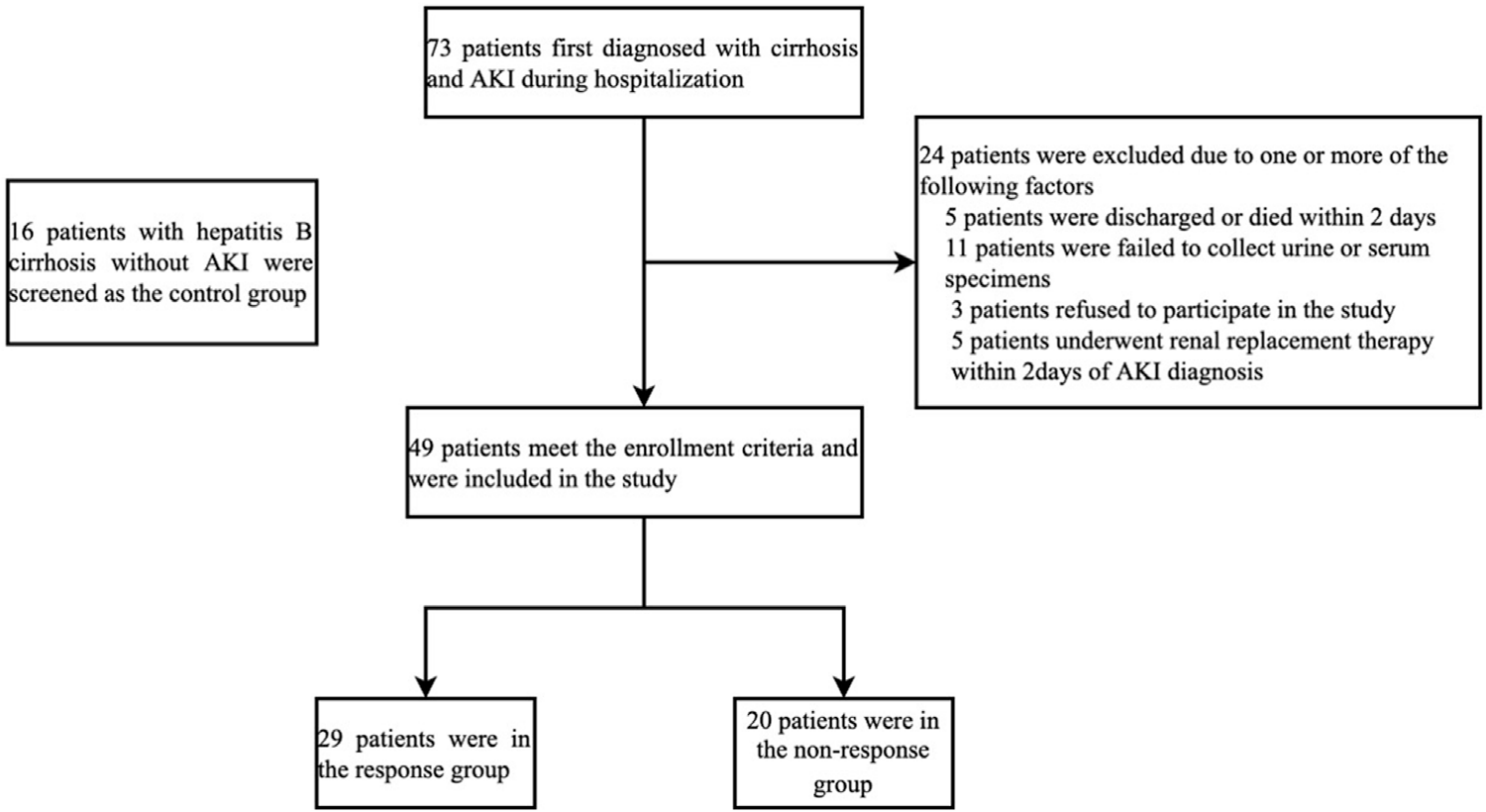
Screening process for patients with hepatitis B cirrhosis and AKI. Abbreviations: Acute kidney injury.

Seventy-three patients with hepatitis B cirrhosis were first diagnosed with AKI during hospitalization. Five patients were discharged or died within 2 days. Five patients underwent renal replacement within 2 days of AKI diagnosis. Three patients refused to participate in the study. And 11 patients were excluded because urine or serum samples could not be collected at diagnosis of AKI. Therefore, 49 patients with newly diagnosed AKI were screened in the study. In addition, 16 hospitalized patients with hepatitis B cirrhosis without AKI at the same period were screened as the control group. The screening process for patients with hepatitis B cirrhosis and AKI is shown in Figure 1. All patients were followed up for 28 days after the diagnosis of AKI and transplant-free survival was recorded. This study complies with the Declaration of Helsinki and has been approved by the Ethics Committee of Youan Hospital Capital Medical University [Jingyou Kelun (2015) No. 19]. All patients signed a written informed consent.

### Definitions

Diagnostic criteria for hepatitis B cirrhosis are based on the Asian-Pacific clinical practice guidelines on the management of hepatitis B (Sarin et al., 2016). AKI is defined according to the International Club of Ascites (ICA) (Angeli et al., 2015): SCr was elevated ≥0.3 mg/d1 (26.5 µmol/L) within 48 h on the basis of cirrhosis or 50% over the baseline SCr obtained in the previous 7 days. The baseline SCr is the stable serum creatinine value available within 7 days prior to the diagnosis of AKI. If a stable baseline value was not available, the SCr value at the time of admission was used as the baseline value (Angeli et al., 2015).

The AKI definition has three stages that indicate the severity of renal dysfunction (Garcia-Tsao et al., 2008). Stage 1 AKI: increase in SCr of more than or equal to 0.3 mg/dl (≥26.5 μmol/L) or increase to more than or equal to 150%–200% from baseline. Stage 2 AKI: increase in SCr to more than 200%–300% from baseline. Stage 3 AKI: increase in SCr to more than 300% from baseline or SCr of more than or equal to 4.0 mg/dl (≥354 μmol/L) with an acute increase of at least 0.5 mg/dl (44 μmol/L). Any patient requiring renal replacement therapy is in stage 3.

### Management of Acute Kidney Injury

Acute Dialysis Quality Initiative (ADQI) and ICA recommend that patients with cirrhosis and AKI discontinue diuretics, vasodilators, and nephrotoxic drugs and give intravenous crystalloids ≥500 ml/day, albumin ≥20 g/day, and plasma 200–400 ml/day for volume expansion (Nadim et al., 2012; Angeli et al., 2015). After the diagnosis of AKI, all patients were given volume expansion therapy consisting of intravenous crystalloids, albumin, and plasma as recommended by ADQI and ICA, with the specific treatment measures given by the clinician depending on the patient’s condition. The efficacy was judged after 48 h of treatment by the aforementioned methods. The response group was defined as a decline in AKI staging or a decrease in SCr ≥0.3 mg/dl from baseline values and no response was defined as no recovery of AKI or progression of the disease.

### Sample Collection and Biomarker Measurement

Demographic and clinical characteristics were collected at the time of diagnosis of AKI. Baseline levels of BUN and SCr were retrospectively collected from patients with cirrhosis and AKI within 7 days prior to the diagnosis of AKI. Meanwhile, demographic and clinical characteristics of patients with hepatitis B cirrhosis in the control group were also collected. In patients with cirrhosis and AKI, a fresh 10 ml urine sample was collected at the time of diagnosis of AKI and 48 h after volume expansion therapy. In the control group, urine sample was randomly collected in the morning. A fresh urine sample was collected in sterile containers, centrifuged at 1000 r/min for 15 min at 2–8°C (Allegra X-22 multifunctional tabletop high-speed centrifuge, 10 cm centrifugal radius), and frozen at −20°C in the refrigerator. All urine samples did not undergo any additional freeze-thaw cycles. Biomarkers in urine were detected by ELISA methods. CUSABIO BIOTECH Co., Ltd. developed assays for KIM-1, NGAL, and IL-18. CMIC HOLDINGS Co., Ltd. developed assays for L-FABP. This assay is based on the Sandwich-ELISA mechanism and enzyme-substrate chromogenic reaction. Experimental reagents were obtained from CUSABIO BIOTECH Co., Ltd. and CMIC HOLDINGS Co., Ltd. All procedure was carried out according to the instructions. All urinary biomarkers were averaged from two measurements.

### Statistical Analysis

The measurement data were expressed as $\bar{x}\pm s$ if they were normally distributed, and the comparison between the two groups was performed using an independent sample Student’s t-test. The measurement data were expressed as median (interquartile range) if they were not normally distributed, and the comparison between the two groups was performed using a Mann–Whitney nonparametric test. The counting data were measured by a chi-square test. Logistic regression was used to analyze the independent risk factors affecting treatment outcome, and the AUC was used to evaluate the predictive efficacy on prognosis. *p* < 0.05 was considered statistically significant, and the statistical software used was SPSS.27.

## Results

### Patients and Characteristics

Among hospitalized patients with hepatitis B cirrhosis, 49 patients with newly diagnosed AKI were screened in the study, and 16 hospitalized patients with hepatitis B cirrhosis without AKI at the same period were screened as the control group. AKI was stage 1 in 38 patients and stage 2–3 in 11 patients at the time of diagnosis of AKI. Baseline demographic, clinical, and biochemical characteristics of patients with and without AKI are shown in Table 1. There were no significant differences in gender, hepatic encephalopathy (HE), age, and infection between patients with and without AKI. Patients who developed AKI had higher white blood cell (WBC), total bilirubin (TBil), albumin (ALB), BUN, SCr, urinary NGAL, urinary IL-18, urinary KIM-1, and urinary L-FABP compared to patients without AKI. Gastrointestinal bleeding (GB) occurred in more patients with AKI than in patients without AKI.

**TABLE1:** Baseline characteristics of patients with and without AKI in cirrhosis.

	AKI (N = 49)	No AKI (N = 16)	*p* Value
Age (year)	55.58 ± 9.32	53.56 ± 7.83	0.439
Gender (male)	36 (73.5%)	13 (81.3%)	0.865
HE	12 (24.49%)	6 (37.5%)	0.491
GB	20 (40.81%)	2 (12.50%)	0.038
Infection	18 (36.73%)	6 (37.50%)	0.956
WBC (×10^9^/L)	8.27 (5.72–11.90)	4.300 (3.25–8.19)	0.002
HB (g/L)	94.00 (69.75–107.00)	91.00 (60.75–127.50)	0.920
PLT (×10^9^/L)	106.00 (52.00–155.00)	72.00 (61.50–128.00)	0.518
NE (%)	76.13 ± 13.21	68.40 ± 16.56	0.062
PTA	57.00 (40.00–74.00)	69.81 ± 20.73	0.087
ALT (U/L)	28.00 (17.17–47.00)	28.00 (18.00–62.00)	0.840
TBil (μmol/L)	60.50 (27.25–315.25)	28.00 (19.75–42.50)	0.006
Albumin (g/L)	30.34 ± 6.38	30.97 ± 5.06	0.010
Na^+^ (mmol/L)	134.06 ± 6.35	138.87 ± 5.85	0.720
Baseline BUN (mmol/L)	8.50 (6.04–13.15)	——	——
Baseline SCr (μmol/L)	85.00 (68.25–102.00)	——	——
BUN (mmol/L)	13.00 (10.00–19.40)	6.55 (4.33–12.00)	0.000^*^
SCr (μmol/L)	134.00 (107.00–162.25)	64.50 (51.50–68.00)	0.000^*^
NGAL (ng/ml)	28.05 (6.46–134.61)	4.64 (2.32–10.84)	0.001
IL-18 (pg/ml)	41.81 (25.22–66.95)	21.28 (14.52–29.97)	0.003
KIM-1 (ng/ml)	1.24 (0.76–2.87)	0.59 (0.38–1.56)	0.008
L-FABP (ng/ml)	12.81 (8.17–21.34)	8.50 (6.05–12.49)	0.047

*p < 0.001.

Abbreviations: AKI, acute kidney injury; HE, hepatic encephalopathy; GB, gastrointestinal bleeding; WBC, white blood cell; HB, hemoglobin; PLT, platelet; NE, neutrophil percentage; PTA, prothrombin activity; ALT, alanine aminotransferase; TBiL, total bilirubin; BUN, blood urea nitrogen; SCr, serum creatinine; NGAL, neutrophil gelatinase-associated lipocalin; IL-18, interleukin-18; KIN-1, Kidney Injury Molecule-1; L-FABP, liver fatty acid-binding protein.

### Urinary Neutrophil Gelatinase-Associated Lipocalin to Predict the Efficacy of Volume Expansion Therapy

**FIGURE 2:**
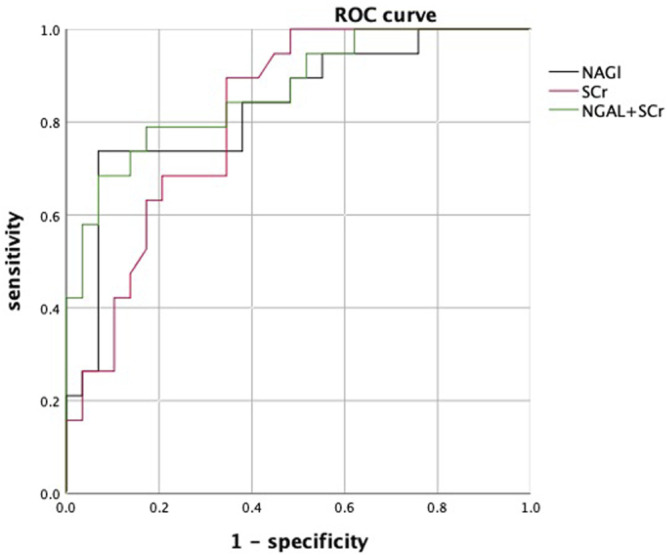
ROC curves of urinary NGAL, SCr, and NGAL + SCr. Abbreviations: ROC: receiver operating characteristic curve; SCr: serum creatinine; NGAL: neutrophil gelatinase-associated lipocalin.

In patients with cirrhosis and AKI, 29 (59.18%) patients were in the response group and 20 (40.81%) patients were in the nonresponse group. At 28 days of follow-up, 25 patients survived and 4 patients died in the response group, while 5 patients survived, and 15 patients died in the nonresponse group. The mortality rate in the nonresponse group was significantly higher than that in the response group (*p* < 0.001). Table 2 shows that WBC, neutrophil percentage (NE), SCr, TBil, urinary NGAL, urinary KIM-1, and urinary L-FABP at diagnosis of AKI were significantly higher in the nonresponse group than in the response group (*p* < 0.05). Prothrombin activity (PTA) was significantly lower in the nonresponse group than in the response group (*p* < 0.05). In multivariate logistic regression analysis, the elevated urinary NGAL and elevated SCr at diagnosis of AKI showed significant association with nonresponse to volume expansion therapy. Using receiver operating characteristic curve (ROC curve), the cutoff values for SCr and urinary NGAL were 128.50 µmol/L and 90.75 ng/ml, respectively (Table3). The AUC of SCr, NGAL, and SCR + NGAL were 0.815, 0.831, and 0.868, respectively, and there was no statistical difference among them (*p* > 0.05) (Figure 2). The changes in urinary biomarkers in patients with cirrhosis and AKI was shown in Supplementary Table S1 and Supplementary Table S2. There was no difference in urinary NGAL before and after treatment in both the response group and the nonresponse group (*p* > 0.05).

**TABLE 2:** Characteristics of patients in the response and nonresponse group to volume expansion therapy in patients with cirrhosis and AKI.

	Response group (N = 29)	Nonresponse group (N = 20)	*p* value
Age (year)	55.45 ± 10.06	55.79 ± 8.38	0.903
Gender (male)	29 (79.3%)	13 (65.03%)	0.609
HE	7 (24.13%)	5 (25%)	1.000
GB	9 (31.05%)	11 (55.04%)	0.093
Infection	10 (34.51%)	8 (40%)	0.694
Deaths	4 (13.8%)	15 (75%)	0.000^*^
WBC (×109/L)	7.56 (4.33–10.52)	9.30 (6.60–17.00)	0.032
HB (g/L)	94.41 ± 28.60	95.00 (62.0–107.0)	0.506
PLT (×10^9^/L)	97.00 (53.00–128.0)	149.00 (51.00–169.00)	0.195
NE (%)	72.02 ± 14.31	82.40 ± 8.28	0.003
PTA	66.27 ± 19.55	40.00 (17.00–68.00)	0.002
ALT (U/L)	27.00 (16.35–36.5)	30.00 (17.00–60.0)	0.548
TBil (μmol/L)	39.0 (20.00–226.0)	99.00 (47.00–455.00)	0.017
Albumin (g/L)	31.37 ± 6.44	28.76 ± 6.11	0.168
Na^+^ (mmol/L)	134.06 ± 5.43	134.05 ± 7.72	0.993
Baseline BUN (mmol/L)	7.50 (5.39–11.70)	9.70 (6.55–19.60)	0.174
Baseline SCr (μmol/L)	75.00 (65.50–93.50)	87.00 (74.00–132.00	0.028
BUN (mmol/L)	12.00 (9.85–18.25)	18.00 (12.00–25.00)	0.161
SCr (μmol/L)	127.71 ± 39.35	176.94 ± 54.62	0.001
NGAL (ng/ml)	12.91 (4.08–42.07)	134.618 (43.20–208.48)	0.000^*^
IL-18 (pg/ml)	37.99 (18.81–66.40)	48.71 (31.90–69.05)	0.393
KIM-1 (ng/ml)	1.06 (0.68–1.69)	2.04 (1.07–3.34)	0.027
L-FABP (ng/ml)	10.37 (7.12–14.93)	20.06 (8.89–45.76)	0.038

*p < 0.001.

Abbreviations: AKI, acute kidney injury; HE, hepatic encephalopathy; GB, gastrointestinal bleeding; WBC, white blood cell; HB, hemoglobin; PLT, platelet; NE, neutrophil percentage; PTA, prothrombin activity; ALT, alanine aminotransferase; TBiL, total bilirubin; BUN, blood urea nitrogen; SCr, serum creatinine; NGAL, neutrophil gelatinase-associated lipocalin; IL-18, interleukin-18; KIN-1, Kidney Injury Molecule-1; L-FABP, liver fatty acid-binding protein.

**TABLE 3:** Cutoff value of different biomarkers in the prediction of response to volume expansion therapy in patients with cirrhosis and AKI.

	AUC	95% CI	Cutoff Value	Sensitivity (%)	Specificity (%)	Youden index	*p* Value
SCr	0.815	0.697–0.933	128.50 µmol/L	89.5	66.5	0.55	<0.001
NGAL	0.831	0.709–0.953	90.75 ng/ml	93.1	66.8	0.668	<0.001
SCr + NGAL	0.868	0.763–0.972	——	78.9	82.8	0.617	<0.001

Abbreviations: SCr, serum creatinine; NGAL, neutrophil gelatinase-associated lipocalin; AUC, area under the receiver operating curve.

### Characteristics of Patients With Cirrhosis at Different Acute Kidney Injury Stages

The characteristics of patients with cirrhosis at different AKI stages was shown in Table 4. We found there was no significant difference in urinary NGAL, urinary IL-18, urinary KIM-1, and urinary L-FABP between the different AKI stages. In AKI stage 1, 25 (65.79%) patients responded to volume expansion therapy. In AKI stage 2–3, 4 (36.36%) patients responded to volume expansion therapy. There was no significant difference in response rate to volume expansion therapy among different AKI periods (*p* = 0.069). There was also no significant difference between 28-day mortality rate and AKI stage at diagnosis (*p* = 0.852). No matter patients in AKI stage 1 or AKI stage 2–3, there was also no significant difference in NGAL, KIM-1, IL-18, and L-FABP before and after treatment (*p* > 0.05) (Supplementary Table S3 and Supplementary Table S4).

**TABLE 4:** Characteristics of patients with cirrhosis at different AKI stages.

	AKI 1 (N = 38)	AKI 2-3 (N = 11)	*p* Value
Age (year)	56.42 ± 9.22	52.40 ± 9.50	0.156
Gender (male)	28 (73.7%)	8 (72.7%)	1.000
Infection	15 (39.5%)	3 (27.3%)	0.701
GB	14 (36.8%)	6 (54.5%)	0.293
HE	11 (28.9%)	1 (9.1%)	0.342
Deaths	15 (39.5%)	4 (36.4%)	0.852
WBC (×109/L)	8.32 (5.53–12.03)	7.67 (5.85–12.20)	0.809
HB (g/L)	93.02 ± 29.67	78.00 (51.50–102.25)	0.228
PLT (×109/L)	109.00 (51.00–155.75)	111.50 (51.75–227.00)	0.559
NE (%)	74.40 ± 13.90	82.73 ± 7.47	0.076
PTA	57.00 (39.75–74.25)	60.50 (35.00–76.75)	0.990
ALT (U/L)	24.50 (14.00–35.25)	58.00 (28.37–108.50)	0.012
TBil (μmol/L)	53.50 (22.50–292.00)	128.50 (38.00–436.25)	0.163
Albumin (g/L)	30.19 ± 6.72	30.91 ± 5.13	0.755
Na^+^ (mmol/L)	135.02 ± 6.53	130.50 ± 4.22	0.045
Baseline BUN (mmol/L)	8.55 (6.17–13.25)	8.50 (2.80–13.30)	0.649
Baseline SCr (μmol/L)	91.73 ± 27.17	71.00 (39.00–97.00)	0.101
BUN (mmol/L)	12.00 (9.95–18.75)	19.20 (9.810–25.25)	0.231
SCr (μmol/L)	127.50 (106.75–155.50)	160.00 (139.75–199.50)	0.040
NGAL (ng/ml)	24.48 (6.59–97.55)	145.64 (5.46–215.78)	0.089
IL-18 (pg/ml)	41.23 (25.01–62.53)	54.88 (33.34–86.06)	0.196
KIM-1 (ng/ml)	1.19 (0.77–2.79)	1.63 (0.70–3.31)	0.458
L-FABP (ng/ml)	12.81 (8.32–18.48)	13.05 (7.59–66.58)	0.677

Abbreviations: AKI, acute kidney injury; HE, hepatic encephalopathy; GB, gastrointestinal bleeding; WBC, white blood cell; HB, hemoglobin; PLT, platelet; NE, neutrophil percentage; PTA, prothrombin activity; ALT, alanine aminotransferase; TBiL, total bilirubin; BUN, blood urea nitrogen; SCr, serum creatinine; NGAL, neutrophil gelatinase-associated lipocalin; IL-18, interleukin-18; KIN-1, Kidney Injury Molecule-1; L-FABP, liver fatty acid-binding protein.

### Predictors of 28-Day Mortality

At 28 days of follow-up, 15 of the 49 patients (30.6%) died, 1 (2.0%) were transplanted, and 3 (6.1%) were discharged due to disease progression. Overall, the probability of transplant-free survival from the diagnosis of AKI was 61.2% at 28 days. In the univariate analysis, the levels of urinary NGAL, KIM-1, and L-FABPA in the nonsurvivors were significantly higher than that in survivors (*p* < 0.05) (Table 5). However, the prognostic significance of urinary NGAL was not observed after the multivariate analysis.

**TABLE 5:** Characteristics of patients categorized according to transplant-free survival at 28 days.

	Survivors (N = 30)	Nonsurvivors (N = 19)	*p* Value
Age (year)	55.14 ± 9.75	52.26 ± 8.85	0.547
Gender (male)	23 (76.7%)	13 (68.4%)	0.524
Infection	9 (30.0%)	3 (27.3%)	0.260
GB	8 (26.7%)	12 (63.2%)	0.011
HE	8 (26.7%)	4 (21.1%)	0.656
WBC(×109/L)	6.60 (4.33–10.21)	11.94 (7.30–18.30)	0.003
HB(g/L)	95.00 (75.50–108.00)	91.00 (64.00–107.00)	0.760
PLT (×109/L)	96.00 (46.50–121.50)	149.00 (81.00–169.00)	0.040
NE, (%)	72.32 ± 14.43	81.96 ± 8.54	0.021
PTA	67.00 (53.00–85.50)	41.00 (17.00–62.00)	0.001
ALT (U/L)	23.00 (14.00–36.00)	33.00 (24.00–77.00)	0.043
TBil (μmol/L)	36.00 (17.50–130.50)	205.00 (55.00–430.00)	0.020
Albumin (g/L)	31.58 ± 6.28	28.45 ± 56.22	0.053
Na+ (mmol/L)	133.93 ± 5.61	134.26 ± 7.51	0.775
BUN(mmol/L)	12.00 (9.85–19.50)	18.00 (11.90–19.40)	0.213
SCr(μmol/L)	120.00 (96.50–151.00)	157.00 (129.00–186.00)	0.008
Baseline NGAL (ng/mL)	12.97 (38.56–73.51)	101.11 (23.62–187.91)	0.004
Baseline IL-18 (pg/mL)	36.41 (19.59–59.89)	54.59 (31.42–69.79)	0.151
Baseline KIM-1 (ng/mL)	1.09 (0.68–1.89)	1.82 (0.92–3.16)	0.044
Baseline L-FABP (ng/ml)	10.21 (7.68–14.77)	21.34 (9.61–48.18)	0.020

Abbreviations: AKI, acute kidney injury; HE, hepatic encephalopathy; GB, gastrointestinal bleeding; WBC, white blood cell; HB, hemoglobin; PLT, platelet; NE, neutrophil percentage; PTA, prothrombin activity; ALT, alanine aminotransferase; TBiL, total bilirubin; BUN, blood urea nitrogen; SCr, serum creatinine; NGAL, neutrophil gelatinase-associated lipocalin; IL-18, interleukin-18; KIN-1, Kidney Injury Molecule-1; L-FABP, liver fatty acid-binding protein.

## Discussion

In this study, we could found that patients with cirrhosis and AKI had higher urinary NGAL, urinary IL-18, urinary KIM-1, and urinary L-FABP compared to patients without AKI. Therefore, biomarkers in urine can also reflect the true situation of kidney damage in patients with cirrhosis. However, in the clinical setting, the diagnosis of AKI is based on changes in SCr concentration according to preestablished criteria (Angeli et al., 2015). Therefore, there is no need for the use of urinary biomarkers to diagnose AKI. By contrast, the usefulness of urinary biomarkers in the field of cirrhosis may be particularly relevant in the differential diagnosis of the type of AKI (Ariza et al., 2015).

We all know that it is important to distinguish between HRS-AKI and ATN in cirrhotic patients, and a large number of studies have emerged in this area. However, in clinical practice, internists also frequently face the challenge of differentiating between PRA from ATN and HRS-AKI when assessing hospitalized patients with AKI. Volume expansion as a therapy can be used for patients with cirrhosis and AKI (Angeli et al., 2015). The volume expansion is not only a treatment but is also used to differentiate PRA from other types of AKI. In this study, after 48 h of volume expansion therapy, 29 patients (59.18%) were effective and 20 patients (40.81%) were ineffective for expansion therapy. It can be found that there are still 40% of patients who do not respond to volume expansion therapy and who may belong to ATN or HRS. From our study, we found that the prognosis is worse in patients who are ineffective in volume expansion therapy, so it is important to take appropriate and timely measures for these patients. However, it usually takes 48 h to determine the effect of volume expansion therapy, which often causes diagnostic delay in patients with cirrhosis and AKI. In patients with ATN, however, patients’ condition would not improve with volume expansion therapy and unnecessary fluid administration might cause harm (Parikh, 2017). Therefore, it is important to find early biomarkers that can predict the efficacy of volume expansion.

SCr is the most used biomarker in patients with renal injury. In this study, SCr was found to be an independent risk factor for nonresponse to volume expansion therapy in patients with cirrhosis and AKI. Using ROC curve, the cutoff value for SCr was 128.50 µmol/L. For patients with SCr >128.50 µmol/L before treatment, they may not respond to expansion therapy and fall under HRS or ATN. However, SCr of patients with cirrhosis is affected by many factors and may not well reflect the real situation of renal injury in patients with cirrhosis (Slocum et al., 2012). Thus, ICA has removed fixed SCr from the definitions of AKI and HRS and pays more attention to the dynamic changes of SCr (Angeli et al., 2015). In addition, SCr is only a marker of kidney filtration, not injury, therefore, SCr is of limited use in identifying types of kidney injury (Verhave et al., 2005). It is difficult to make a determination of the pathological type of AKI by fixed levels of SCr alone.

Some studies found urinary KIM-1, urinary NGAL, urinary IL-18, and urinary L-FABP are helpful to distinguish whether AKI is organic or functional damage in patients without cirrhosis (Sorof, 1994; Ichimura et al., 1998; Parikh et al., 2004; Barreto et al., 2014; Belcher et al., 2014; Puthumana et al., 2017; Hamdy et al., 2018; Jaques et al., 2019). NGAL is a member of the lipocalin protein family and was first discovered by Kjeldsen et al. in 1993 while studying human neutrophil gelatinase B (Kjeldsen et al., 1993). Under normal physiological conditions, NGAL is expressed in the kidney at very low levels, but in cases of renal ischemia or acute nephrotoxic damage, NGAL can be expressed in large amounts in the renal tubules and elevated urinary NGAL can be undetected in the urine during the early stages of renal injury (Singer et al., 2013). Some studies found that urinary NGAL can be used to distinguish ATN from PRA and HRS, and it is also an independent predictor of poor prognosis in patients with liver cirrhosis (Barreto et al., 2014; Belcher et al., 2014; Puthumana et al., 2017; Hamdy et al., 2018; Jaques et al., 2019),and a study found that urinary NGAL can be used to distinguish PRA from ATN in patients without cirrhosis (Nickolas et al., 2008). However, NGAL has rarely been used to differentiate PRA from ATN and HRS in cirrhotic patients. In this study, urinary NGAL was an independent risk factor for nonresponse to volume expansion therapy in patients with cirrhosis and AKI, and the cutoff value for urinary NGAL was 90.75 ng/ml. Patients with urinary NGAL<90.75 ng/ml can be treated with aggressive volume expansion therapy, but patients with cirrhosis and AKI with urinary NGAL>90.75 ng/ml before treatment may not respond to volume expansion therapy. The AUC of urinary NGAL is 0.831 and is higher than the AUC of SCr which is 0.815. In addition, a combination of the two biomarkers cannot improve the accuracy. The urinary NGAL can be used as a new early warning indicator to predict the effectiveness of volume expansion therapy in patients with cirrhosis and AKI. In other words, measurement of urinary NGAL in patients with cirrhosis prior to treatment can be used earlier to identify PRA from ATN and HRS in patients with cirrhosis than traditional identification methods. And patients with high level of urinary NGAL also have a poor prognosis.

In the past, HRS is classically considered as functional kidney injury. However, in our study, another finding was that the urinary NGAL is also increased in patients with HRS-AKI and ATN compared to PRA. The levels of NGAL increased in HRS-AKI suggest that HRS-AKI may also include some degree of structural injury. Similar to our study, some studies also found that the levels of urinary NGAL in patients with HRS were significantly higher than in those with PRA and lower than in those with ATN (Belcher et al., 2014). Thus, HRS and ATN can also be viewed as a continuous process, rather than two separate diseases. In a study, among 18 patients with diagnosis of HRS-CKD, renal histology revealed chronic tubular interstitial injury in 13, acute tubular interstitial injury in 12, glomerular injury in 10, and vascular injury in 12 (Trawalé et al., 2010). Although there is no obvious clinical sign of structural kidney injury, HRS patients may still have structural injury at the pathological level. Therefore, although there are many difficulties, it is necessary to obtaining and assessing kidney biopsy specimens in patients with cirrhosis to create a kidney tissue atlas. Some studies have found that urinary NGAL is associated with short-term survival in patients with cirrhosis and AKI (Huelin et al., 2019). However, urinary NGAL did not show good predictive accuracy in our study in predicting short-term survival. This finding is significantly different from previous studies evaluating the prognostic value of NGAL, possibly due to the relatively low number of patients included.

The study also has the following limitations: First, the sample size of the study is small, and the observation time is short; thus, the sample size still needs to be further expanded. Second, as an observational study, treatment of patients was not standardized and thus we could not well assess the relationship between biomarker levels and treatment response. However, current guidelines have not identified urine markers with high sensitivity and specificity to distinguish different types of kidney injury, especially in distinguishing PRA from ATN and HRS. Therefore, our prospective study may provide some evidence-based data for future guideline writing.

## Conclusion

Elevated urinary NGAL is an independent risk factor for nonresponse to volume expansion therapy, and the results of the study indicate that urinary NGAL levels are highly accurate in the differential diagnosis between PRA and other types of AKI in patients with hepatitis B cirrhosis. The increased levels of urinary NGAL in HRS also suggest that HRS may also include some degree of structural injury. Finally, urinary NGAL has clinical potential to influence the treatment of AKI in cirrhosis and it deserves further study in patients with cirrhosis and AKI.

## Data Availability

The original contributions presented in the study are included in the article/Supplementary Material, further inquiries can be directed to the corresponding author.
