# Supplementary material for: Urinary Neutrophil Gelatinase-Associated Lipocalin Can Predict the Efficacy of Volume Expansion Therapy in Patients With Hepatitis B Cirrhosis and AKI
Source: Front Pharmacol. 2022 Jun 15;13:839250. doi: 10.3389/fphar.2022.839250 (PMC9240615; doi:10.3389/fphar.2022.839250)
Supplement: Supplementary file 1 [file Table1.DOCX]

**Table S1**

**Comparison of urinary biomarkers in response group before and after treatment**

| Response group (N=29) | before treatment | After treatment | *P* value |
| --- | --- | --- | --- |
| NGAL (ng/mL) | 12.91 (4.08-42.07) | 13.12 (5.07-36.63) | 0.869 |
| IL-18 (pg/mL) | 37.99 (18.81-66.40) | 34.81 (18.53-69.87) | 0.657 |
| KIM-1 (ng/mL) | 1.06 (0.68-1.69) | 0.88 (0.47-1.63) | 0.112 |
| L-FABP (ng/mL) | 10.37 (7.12-14.93) | 9.92 (6.55-14.99) | 0.354 |

Abbreviations: NGAL: neutrophil gelatinase–associated lipocalin; IL-18: interleukin-18; KIN-1: Kidney Injury Molecule-1; L-FABP: Liver Fatty acid binding protein.
