# Supplementary material for: Urinary Neutrophil Gelatinase-Associated Lipocalin Can Predict the Efficacy of Volume Expansion Therapy in Patients With Hepatitis B Cirrhosis and AKI
Source: Front Pharmacol. 2022 Jun 15;13:839250. doi: 10.3389/fphar.2022.839250 (PMC9240615; doi:10.3389/fphar.2022.839250)
Supplement: Supplementary file 2 [file Table2.DOCX]

**Table S2**

**Comparison of urinary biomarkers in non-response group before and after treatment**

| Non-response group (N=20) | Before treatment | After treatment | *P* value |
| --- | --- | --- | --- |
| NGAL (ng/mL) | 134.61 (43.20-208.48) | 164.43 (66.21-42.04) | 0.438 |
| IL-18 (pg/mL) | 48.71 (31.90-69.05) | 49.03 (27.24-81.98) | 0.469 |
| KIM-1 (ng/mL) | 2.04 (1.07-3.34) | 3.38 (1.49-5.15) | 0.013 |
| L-FABP (ng/mL) | 20.06 (8.89-45.76) | 20.11 (10.01-60.72) | 0.460 |

Abbreviations: NGAL: neutrophil gelatinase–associated lipocalin; IL-18: interleukin-18; KIN-1: Kidney Injury Molecule-1; L-FABP: Liver Fatty acid binding protein.
