# Supplementary material for: Urinary Neutrophil Gelatinase-Associated Lipocalin Can Predict the Efficacy of Volume Expansion Therapy in Patients With Hepatitis B Cirrhosis and AKI
Source: Front Pharmacol. 2022 Jun 15;13:839250. doi: 10.3389/fphar.2022.839250 (PMC9240615; doi:10.3389/fphar.2022.839250)
Supplement: Supplementary file 3 [file Table3.DOCX]

**Table S3**

**Comparison of urinary markers in patients with AKI stage 1 before and after treatment**

| AKI 1 (N=38) | Before treatment | After treatment | *P* value |
| --- | --- | --- | --- |
| NGAL (ng/mL) | 24.48 (6.59-97.55) | 21.78 (9.01-12.49) | 0.330 |
| IL-18 (pg/mL) | 41.23 (25.01-62.53) | 47.71 (24.60-73.69) | 0.288 |
| KIM-1 (ng/mL) | 1.19 (0.77-2.79) | 1.15 (0.61-3.49) | 0.586 |
| L-FABP (ng/mL) | 12.81 (8.32-18.48) | 10.22 (7.45-18.23) | 0.432 |

Abbreviations: NGAL: neutrophil gelatinase–associated lipocalin; IL-18: interleukin-18; KIN-1: Kidney Injury Molecule-1; L-FABP: Liver Fatty acid binding protein.
