# Supplementary material for: Urinary Neutrophil Gelatinase-Associated Lipocalin Can Predict the Efficacy of Volume Expansion Therapy in Patients With Hepatitis B Cirrhosis and AKI
Source: Front Pharmacol. 2022 Jun 15;13:839250. doi: 10.3389/fphar.2022.839250 (PMC9240615; doi:10.3389/fphar.2022.839250)
Supplement: Supplementary file 4 [file Table4.DOCX]

**Table S4**

**Comparison of urinary markers in patients with AKI stage 2-3 before and after treatment**

| AKI 2-3 (N=11) | Before treatment | After treatment | *P* value |
| --- | --- | --- | --- |
| NGAL (ng/mL) | 145.64 (5.46-215.78) | 129.03 (29.71-210.30) | 0.678 |
| IL-18 (pg/mL) | 54.88 (33.34-86.06) | 46.80 (16.91-104.78) | 0.110 |
| KIM-1 (ng/mL) | 1.63 (0.70-3.31) | 1.56 (0.77-4.03) | 0.859 |
| L-FABP (ng/mL) | 13.05 (7.59-66.58) | 14.24 (9.13-66.19) | 0.374 |

Abbreviations: NGAL: neutrophil gelatinase–associated lipocalin; IL-18: interleukin-18; KIN-1: Kidney Injury Molecule-1; L-FABP: Liver Fatty acid binding protein.
